# The clinical and prognostic implications of pluripotent stem cell gene expression in hepatocellular carcinoma

**DOI:** 10.3892/ol.2013.1151

**Published:** 2013-01-23

**Authors:** XIN YIN, YI-WEI LI, JIAN-JUN JIN, YIN ZHOU, ZHENG-GANG REN, SHUANG-JIAN QIU, BO-HENG ZHANG

**Affiliations:** 1 Liver Cancer Institute and Zhongshan Hospital, Key Laboratory of Carcinogenesis and Cancer Invasion, Ministry of Education, Shanghai 200032, P.R. China; 2 Experimental Center of Zhongshan Hospital, Fudan University, Shanghai 200032, P.R. China

**Keywords:** hepatocellular carcinoma, pluripotent stem cell genes, prognosis, curative liver resection

## Abstract

Recently, growing evidence has demonstrated that aberrant expression of pluripotent stem cell-related genes may confer primitive and aggressive traits and be associated with unfavorable clinical outcomes in certain solid cancers. However, the role of pluripotent stem cell gene expression in hepatocellular carcinoma (HCC) remains unexplored. We evaluated the expression of the pluri potent stem cell genes Oct4, Sox2 and Klf4, as well as that of the c-Myc, Nanog and Lin28 genes in HCC samples and corresponding adjacent non-tumor liver samples obtained from 57 patients using quantitative real-time reverse transcription-PCR (qRT-PCR). The results revealed that six pluripotent stem cell gene expression levels were upregulated in the tumor tissues compared with the corresponding adjacent non-tumor liver tissues. In HCC tissues, aberrant expression of Sox2 and Lin28 was associated with a large tumor size (P=0.02 and P=0.03, respectively), while increased expression levels of c-Myc (P=0.01) were correlated with vascular invasion. Moreover, high Klf4 expression levels were associated with aggressive tumor behaviors in terms of vascular invasion (P=0.02) and poor tumor differentiation (P=0.03). Survival analysis revealed that Klf4 expression was independently associated with overall survival [OS; hazard ratio (HR), 8.61; 95% confidential interval (CI), 2.7–27.5; P<0.001] and recurrence-free survival (RFS; HR, 3.96; 95% CI, 1.3–11.6; P=0.01). In conclusion, pluripotent stem cell genes are associated with HCC progression and a poor prognosis. The development of therapeutic strategies, including adjuvant therapy, that take cancer stem cell (CSC)-related markers into consideration is likely to be a key factor in further improvements of the prognosis of HCC patients undergoing curative liver resection.

## Introduction

Hepatocellular carcinoma (HCC) is one of the most common and aggressive types of cancer worldwide. Its highly invasive and metastatic phenotypes are the key reasons for treatment failure and a poor prognosis. Despite advances in diagnostic and therapeutic techniques, a high failure rate and a low median survival rate are currently observed in patients following multimodality therapies with recurrent, intractable HCC. To improve patient survival, it is important to elucidate the regulatory mechanisms that control the tumor-initiating and metastatic properties of HCC.

Cancer stem cells (CSCs), also defined as tumor-initiating stem-like cells (TISCs), are a subpopulation of neoplastic cells that possess characteristics of normal stem cells, most notably the ability to self-renew and to differentiate into heterogeneous non-tumorigenic cancer cells that comprise the bulk of the tumor. There has been abundant evidence that supports the existence of cancer stem cells in HCC (1). Under the CSC hypothesis, factors contributing to a poor prognosis, including resistance to conventional therapies, tumor recurrence and metastasis, may be associated with a small subset of highly tumorigenic stem-like cells (2). However, despite growing evidence for the existence of tumor-initiating cells, the stem cell properties of tumor-initiating cells remain largely unresolved (3,4). Studies have suggested that CSCs have common molecular signatures that are similar to those of pluripotent embryonic stem cells (5,6). The core transcription factors of this stem cell-like signature, including Oct4, Sox2, Klf4, c-Myc, Nanog and Lin28, have been used to successfully reprogram differentiated somatic cells into pluripotent stem cells (7,8). Studies have also revealed that the expression of stem cell-related genes may help to distinguish tumors associated with distinct clinical outcomes in certain solid types of cancer, such as medulloblastoma and colorectal cancer (9,10). However, thus far, a possible correlation between pluripotent stem cell genes and the prognosis has not been established in HCC. Our study aimed to evaluate the expression profiles of the pluripotent stem cell genes (Oct4, Sox2, Klf4, c-Myc, Nanog and Lin28) using quantitative reverse transcription (qRT)-polymerase chain reaction (PCR), and to determine its possible prognostic significance in HCC.

## Materials and methods

### Patients and sample collection

A total of 57 pairs of HCC tissues and adjacent non-tumor liver tissues were obtained from patients who underwent curative liver resection consecutively in a single group at the Department of Hepatic Surgery, Zhongshan Hospital, Fudan University from January, 2006 to December, 2007. Tissue samples were frozen in liquid nitrogen immediately following resection and were stored at −80°C until RNA extraction was performed. Patient selection was according to the following inclusion criteria: i) confirmed pathological diagnosis of HCC; ii) without anticancer treatments and distant metastases prior to surgery; iii) undergoing curative liver resection for HCC, defined as macroscopically complete removal of the tumor as described previously by Sun *et al*(11); and iv) availability of fresh tissue specimens and their complete clinical data. The patients included 48 males (84.2%) and 9 females (15.8%), with a median age of 49 years (range, 33–67 years). Fifty-four patients (94.7%) were positive for hepatitis B virus. Tumor size ranged from 1.5 to 14.0 cm, with a median size of 6.0 cm. All tumors were histologically diagnosed as HCC with Edmondson grade I in 2 cases, grade II in 47 cases and grade III in 8 cases. The tumor stages were classified according to the 7th edition tumor-node-metastasis (TNM) classification of the International Union Against Cancer. Twenty-four cases were classified as stage I, 25 as stage II and 8 as stage III. The study was performed in accordance with the ethical standards of the Declaration of Helsinki and was approved by the Ethics Committee of Zhongshan Hospital, Fudan University. Informed consent was obtained from all patients participating in the present study.

### Real-time quantitative PCR analysis

Total RNA was extracted from HCC tissues and adjacent non-tumor liver tissues using TRIzol (Invitrogen Life Technologies; Carlsbad, CA, USA) according to the manufacturer’s instructions. Total RNA (2 *μ*g) was reverse transcribed using the Primescript RT reagent kit (Takara Bio, Inc.; Tokyo, Japan). Expression levels of Oct4 (NM_203289), Sox2 (NM_003106) and Klf4 (NM_004235), as well as the c-Myc (NM_002467), Nanog (NM_002467) and Lin28 (NM_024674) genes were evaluated by quantitative real-time PCR. Real-time PCR for quantification was performed using SYBR Premix Ex Taq (Takara Bio, Inc.). The reactions were performed in triplicate. The expression levels of target genes were normalized to the expression level of GAPDH (NM_002046), a housekeeping gene control. Primer sequences of target genes and GAPDH were as follows: forward: 5’-GAGAAGGATGTGGTCCGAGTGTG-3’ and reverse: 5’-GGCAGATGGTCGTTTGGCTGAATA-3’ for human Oct4; forward: 5’-CGCCCCCAGCAGACTTCACA-3’ and reverse: 5’-CTCCTCTTTTGCACCCCTCCCATTT-3’ for human Sox2; forward: 5’-AGAGGAGCCCAAGCCAAAGAG-3’ and reverse: 5’-CGAATTTCCATCCACAGCCGTC-3’ for human Klf4; forward: 5’-CAGAGTGCATCGACCCCTCG-3’ and reverse: 5’-TTCCTCCTCAGAGTCGCTGC-3’ for human c-Myc; forward: 5’-TGAACCTCAGCTACAAACAGGTG-3’ and reverse: 5’-AACTGCATGCAGGACTGCAGAG-3’ for human Nanog; forward: 5’-CTCCGTGTCCAACCAGCAG-3’ and reverse: 5’-CACGTTGAACCACTTACAGATGC-3’ for human Lin28; forward: 5’-AGCCACATCGCTCAGACA-3’ and reverse: 5’-GCCCAATACGACCAAATCC-3’ for human GAPDH. The relative genomic expression was calculated by 2^−ΔΔCt^, as previously described by Schmittgen and Livak (12).

### Follow-up

Patients were followed up until December, 2011 and the median follow-up period was 22 months (range, 5–58 months). Follow-up procedures were performed according to a uniform guideline previously established in our institute (13). Briefly, patients were monitored by abdominal ultrasonography, serum α-fetoprotein (AFP) levels and chest radiography with an interval of 2–4 months following discharge. When a recurrence was suspected, computed tomography (CT) scanning or magnetic resonance imaging (MRI) was performed immediately. Treatment modalities following relapse were administered according to a uniform guideline by Sun *et al*(14). Overall survival (OS) was defined as the time interval between the date of surgery and the date of mortality or the last observation time. For surviving patients, the data were censored at the last follow-up. Recurrence-free survival (RFS) was defined as the time interval between the date of surgery and the date of diagnosis of any type of tumor relapse (intrahepatic recurrence and extrahepatic metastasis).

### X-tile analysis

As there are no established cut-off points available for pluripotent stem cell gene expression in HCC, X-tile analysis (X-tile software, version 3.6.1; Yale University School of Medicine; New Haven, CT, USA) was performed to generate an optimal cut-off point for categorization of the stem cell gene expression levels. The X-tile program split the cohort randomly into a matched training and validation set as a method for selecting an optimal cut-off point. It calculated a P-value for every possible division of the cohort expression data. A two-dimensional graph with its corresponding survival curves was plotted, where each colored pixel was proportional to its χ^2^ value. The program automatically calculated the maximum χ^2^ value, which served as a cut-off point that predicted the prognosis (15).

### Statistical analysis

Numerical data are presented as mean ± standard deviation or as median (range). Quantitative data were compared using a Wilcoxon signed rank sum test. Categorical data were analyzed by the χ^2^ or Fisher exact tests as appropriate. A Spearman’s correlation test was applied to analyze the correlations. OS and RFS curves were generated using the Kaplan-Meier method, and the differences between curves were assessed by the log-rank test. Independent prognostic factors were estimated by the Cox proportional hazards stepwise regression model. The Statistical Package for the Social Sciences (SPSS) 13.0 for Windows (SPSS, Inc.; Chicago, IL, USA) was the software used for assessment. A two-tailed P<0.05 was considered to indicate a statistically significant difference.

## Results

### Pluripotent stem cell gene expression in HCC tissues

**Figure 1:**
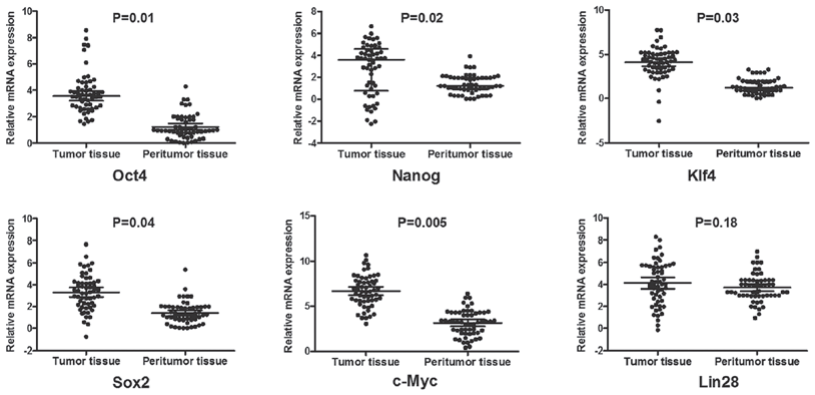
Real-time quantitative polymerase chain reaction (PCR) analysis of six pluripotent stem cell genes in hepatocellular carcinoma (HCC) tissue and adjacent non-tumor liver tissue. The results demonstrated a higher expression of Oct4, Sox2, Klf4, c-Myc and Nanog mRNA in HCC tissues compared with non-tumor liver tissues (P<0.05 for all genes tested).

The expression of six pluripotent genes was evaluated in 57 HCC patients through qPCR analysis. The results revealed that the expression levels of Oct4, Sox2, Klf4, c-Myc and Nanog in HCC specimens were signifcantly higher than those in the corresponding adjacent non-tumor tissues (Fig. 1). Analysing all possible combinations of transcription factors demonstrated that a significant correlation was only achieved between Oct4 and Nanog (Spearman’s correlation coefficient, 0.44; P<0.001), suggesting a possible functional link between Oct4 and Nanog in HCC cases.

### Selection of optimal cut-off values

**Figure 2:**
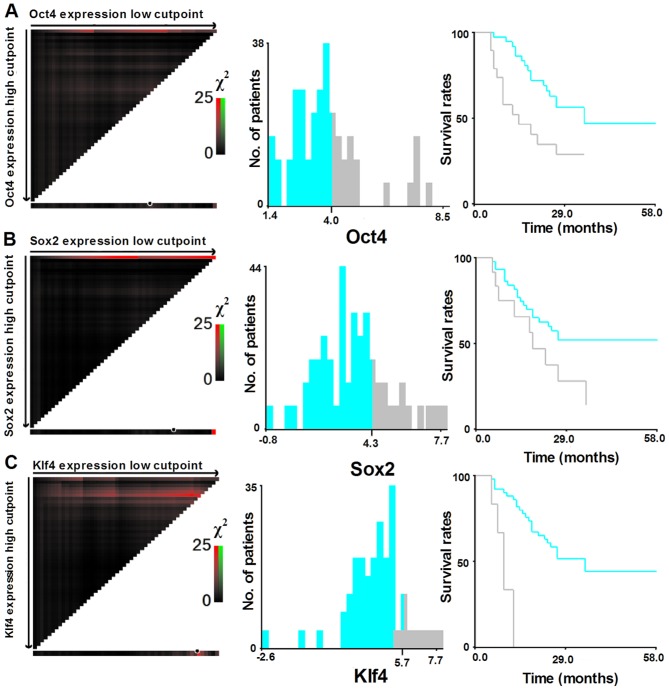
X-tile analysis of the prognostic significance of Oct4, Sox2 and Klf4 in HCC patients. The plot shows the χ^2^ log-rank values created when the cohort was divided into a matched training and validation set. The cut-off point highlighted by the black/white circle in the left panels is demonstrated in a histogram (middle panels) and a Kaplan-Meier plot (right panels). The optimal cut-off point for each gene was defined by the most significant base in the X-tile analysis, and are as follows: (A) 4.0 for Oct4 (P_min_=0.02); (B) 4.3 for Sox2 (P_min_=0.05); and (C) 5.7 for Klf4 (P_min_<0.001).

**Figure 3:**
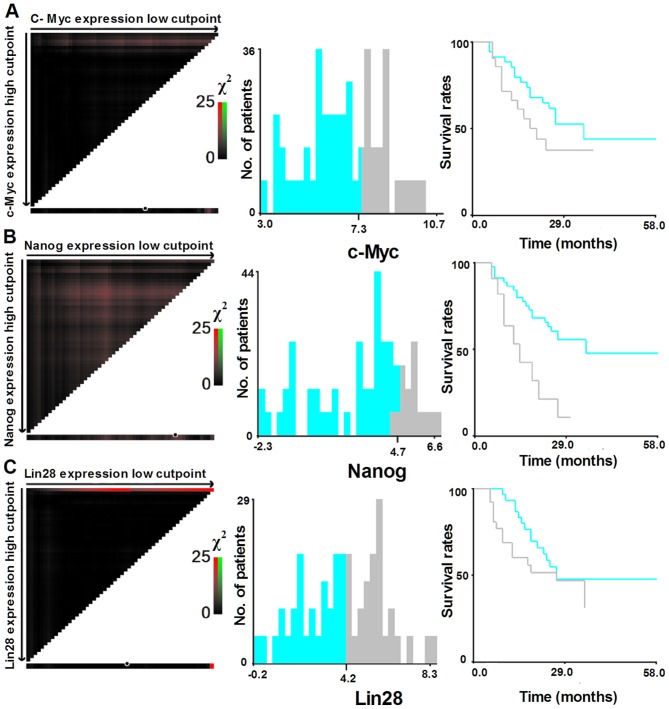
X-tile analysis of the prognostic significance of c-Myc, Nanog and Lin28 in HCC patients. The optimal cut-off point for each gene was defined by the most significant base in the X-tile analysis, and are as follows: (A) 7.3 for c-Myc (P_min_=0.26); (B) 4.7 for Nanog (P_min_=0.01); and (C) 4.2 for Lin28 (P_min_=0.38).

To assess the statistical significance and to avoid the problems of multiple cut-off point selection, the X-tile program was employed to determine the cut-off values for the expression of six pluripotent genes. According to the X-tile program, the patient cohort was divided into low expression and high expression populations based on a cut-off point of 4.0 for Oct4 (P_min_=0.02), 4.3 for Sox2 (P_min_=0.05), 5.7 for Klf4 (P_min_<0.001), 4.7 for Nanog (P_min_=0.01) 7.3 for c-Myc (P_min_=0.26) and 4.2 for Lin28 (P_min_=0.38) (Figs. 2 and 3).

### Correlation of pluripotent stem cell gene expression with clinicopathologic features

As shown in Table I, the expression levels of the Sox2 and Lin28 genes were significantly correlated with tumor size. The mean Sox2 and Lin28 expression levels in the 28 cases with a large tumor size (>5 cm) were 3.79±0.29 and 4.66±0.40, respectively; whereas in the 29 cases with a small tumor size, the levels were 2.79±0.29 and 3.55±0.31 (P=0.02 and 0.03), respectively. With respect to vascular invasion, marked increases in Klf4 (P=0.02) and c-Myc (P=0.01) levels were detected in HCC patients with vascular invasion. With regard to tumor differentiation, a significant correlation was identified between Klf4 expression and poor tumor differentiation (P=0.03). However, no statistical difference was identified in the expression of pluripotent genes when compared with other clinicopathological factors, including age, gender, hepatitis B surface antigen (HBsAg), AFP, liver cirrhosis, tumor number, tumor encapsulation and TNM stage.

### Involvement of pluripotent stem cell gene expression in the prognosis of HCC

**Figure 4:**
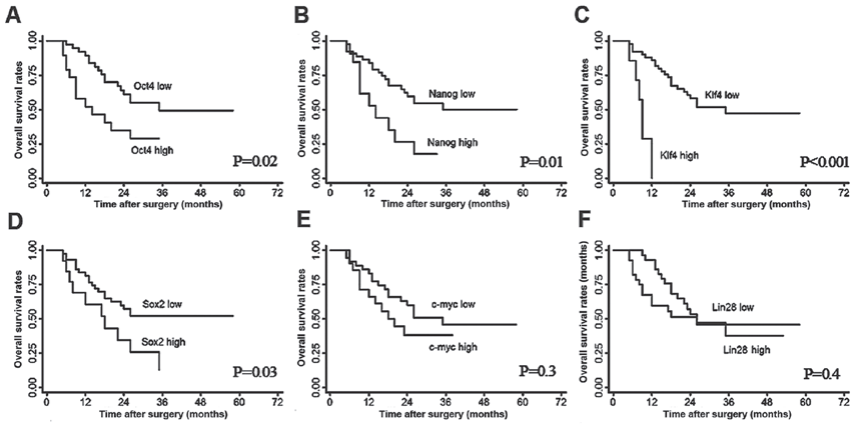
Overall survival (OS) as assessed by Kaplan-Meier analysis in hepatocellular carcinoma (HCC) patients according to pluripotent stem cell gene expression. High levels of Oct4, Klf4 and Nanog mRNA expression were significantly correlated with a poorer OS (P= 0.01, P= 0.02 and P<0.01, respectively).

**Figure 5:**
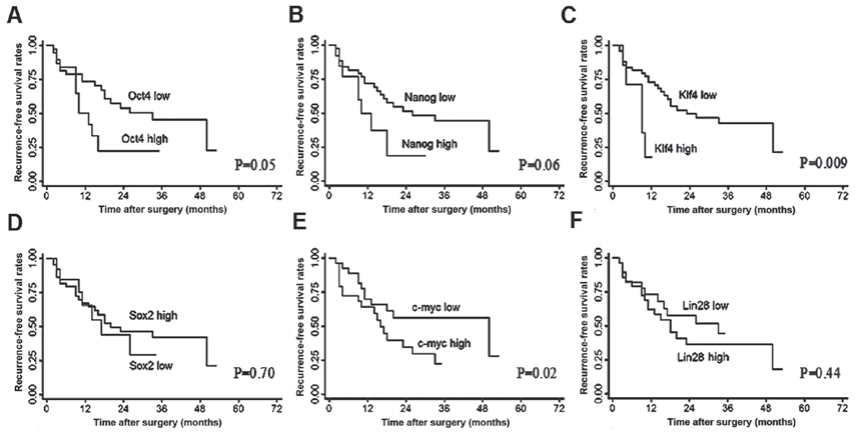
Recurrence-free survival (RFS) as assessed by Kaplan-Meier analysis in hepatocellular carcinoma (HCC) patients according to pluripotent stem cell gene expression. A high level of Klf4 mRNA expression was significantly correlated with a poorer RFS (P=0.009).

The OS and RFS rates for the whole study population were 76.9 and 66.9% at 1 year, 42.4 and 36.7% at 3 years, as well as 42.4 and 19.7% at 5 years, respectively. Upon univariate analysis, Oct4, Klf4 and Nanog mRNA expression were correlated with an unfavorable OS (Fig. 4A–C). High Klf4 expression was also correlated with poor RFS compared with low Klf4 expression (median, 9.0 vs. 15.5 months; P=0.009; Fig. 5C). The multivariate analysis revealed that Klf4 expression was independently correlated with OS (HR, 8.61; 95% CI, 2.7–27.5; P<0.001) and RFS (HR, 3.96; 95% CI, 1.3–11.6; P=0.01). Additionally, vascular invasion and complete tumor encapsulation were also independent predictors for OS (P=0.006 and 0.04, respectively). Moreover, tumor number and TNM stage were observed to be independently correlated with RFS (P=0.04 and P=0.03, respectively).

## Discussion

Although the number of HCC cases was limited in our study, the results were intriguing. We demonstrated that positive expression of Klf4 was significantly correlated with vascular invasion, poor tumor differentiation, short post-operative recurrence time and a poor prognosis.

Klf4, previously known as gut-enriched Krüppel-like factor (GKLF), is highly expressed in the post-mitotic cells of the gut and skin. Klf4 was first identified as a tumor suppressor, owing to frequent loss of Klf4 expression in gastric, colon, esophageal, bladder and lung cancer, as well as in pancreatic ductal carcinoma (16–18). However, subsequent studies proposed the expression of Klf4 was upregulated in oral squamous carcinoma, primary breast ductal carcinoma and human skin squamous cell carcinoma, and correlated with carcinogenesis and tumor progression (19–21). These studies suggested that Klf4 may function as either a tumor suppressor or an onco-gene, depending on the tumor type. To date, the role of Klf4 in HCC carcinogenesis and progression remains unclear. Our study indicated that the expression of Klf4 mRNA was significantly correlated with vascular invasion and poor tumor differentiation. Notably, positive expression of Klf4 mRNA was also correlated with tumor relapse and a poor prognosis in patients with HCC. It is difficult to explain the effects of Klf4 on HCC invasion and progression, and there is no direct evidence to indicate this clearly *in vivo* and *in vitro*. Further study is required to determine the precise role of KLF4 in HCC and in other human cancer types.

Sox2, along with Oct4 and Nanog, plays a crucial role in the maintenance of embryonic stem cell pluripotency (22). Previous studies have demonstrated that Sox2 is also involved in promoting tumorigenesis, proliferation, and dedifferentiation of human lung squamous cell carcinoma (23) and breast cancer (24). Reduced Sox2 levels in glioblastoma tumor-initiating cells can cause proliferation to cease and a loss of tumorigenicity (25), while overexpression of Sox2 in pancreatic cancer is correlated with an invasive and metastatic phenotype (26). To elucidate the role of Sox2 in HCC, we investigated the correlation between Sox2 mRNA expression and clinicopathological variables associated with tumor progression. The results suggested that Sox2 mRNA is upregulated in HCC tissue, compared with in the adjacent non-tumor tissue and is correlated with a large tumor size. The survival analysis further indicated that patients with a high level of Sox2 had a short survival time (25 months) compared with those with a low level of Sox2 (17 months); however, the difference was not significant (P=0.05). These results suggested that the upregulation of Sox2 may play an important oncogenic role in HCC and represent an acquired malignant proliferative phenotypic feature of tumor cells.

Oct4, a transcription factor in the POU protein family, expressed in both embryonic and adult stem cells, has been proposed to be associated with the pluripotency, proliferative potential and self-renewal properties of embryonic stem cells (ESCs) and germ cells (27). Nanog, a downstream target of Oct4 that contributes to cell fate determination of the pluripotent inner cell mass during embryonic development, is also specifically expressed in human embryonic pluripotent stem cells (28). The expression of Oct4 and Nanog is usually confined to pluripotent cells of the developing embryo. Studies have suggested that Oct4 and Nanog may participate in the tumorigenicity and tumor progression of various types of cancer, including breast cancer, glioma, human endometrial adenocarcinoma, gastric cancer and colorectal cancer (29–33). Coexpression of Oct4 and Nanog has also been identified to be correlated with pancreatic carcinogenesis as well as with a poor prognosis of oral squamous cell carcinoma patients (34,35). In the present study, we found that Oct4 and Nanog expression levels were elevated in HCC tissue compared with the adjacent non-tumor liver tissue. Moreover, Oct4 mRNA expression was positively correlated with Nanog mRNA expression (Spearman’s correlation coefficient, 0.44; P<0.001). Patients with a positive expression of Oct4 or Nanog had a relatively low survival rate (Fig. 3A and B). These results indicated that Oct4 and Nanog, the two master transcription factors, which are correlated with stem cell self-renewal and differentiation, may also be correlated with HCC carcinogenesis and the unfavorable prognosis of HCC patients following surgery.

The Myc transcription factor is one of the most important somatically mutated oncogenes in human cancer. There is increasing evidence to support the role of the c-Myc protooncogene in tumor onset and progression. The Myc protein is able to confer a selective advantage in cancer cells by promoting proliferation, cell survival, differentiation blockade, genetic instability and angiogenesis, all of which may indirectly contribute to metastasis (36–38). Upregulating c-Myc expression drives cell growth and vasculogenesis, reduces cell adhesion and promotes metastasis (39). c-Myc also induces the production of the vascular endothelial growth factor (VEGF) by tumor cells, leading to tumor vasculo genesis in pancreatic islet cancer and non-small cell lung cancer (38,40). In accordance with these studies, our data also demonstrated that an abnormal expression of c-Myc mRNA in HCC tissue was correlated with vascular invasion, which suggested an important role of c-Myc in HCC vasculogenesis.

The expression of Lin28 has been demonstrated to promote oncogenesis and participate in the tumor progression of cancer. Dangi-Garimella *et al*(41) proposed that upregulated Lin28 expression accelerated metastasis in breast cancer, as Lin28 repressed let-7 processing that in turn inhibited HMGA2, a chromatin remodeling protein that activated pro-invasive and pro-metastatic genes. Saiki *et al*(10) and Hamano *et al*(42) demonstrated that Lin28 expression was closely correlated with cancer progression in colorectal and oesophageal cancer. However, the current study did not yield definitive findings with respect to the potential role of Lin28 genes in HCC carcinogenesis, aggressiveness and a poor prognosis. The expression of Lin28 mRNA was slightly higher in HCC tissue than in the adjacent tumor tissue (P=0.18). No correlation was observed between Lin28 expression and HCC patient survival, post-operative recurrence or prognosis.

In summary, the results of our comprehensive analysis of six pluripotent stem cell genes in 57 HCC cases suggest that upregulated expression of pluripotency genes in HCC cells may be important in HCC carcinogenesis. Altered expression of Klf4, Sox2, Oct4, Nanog and c-Myc genes indicates the abundance of cancer stem-like cells (CSLCs) in primary tumors that may be key resources for tumor progression and the poor prognosis of HCC. As there are no relevant therapeutic strategies to specifically target cells with a CSC-like phenotype in HCC, further investigations to establish the appropriate adjuvant strategies are required.

## Figures and Tables

**Table I:** Correlation between pluripotent stem cell gene expression and clinicopathological variables in hepatocellular carcinoma (HCC) patients.

	Oct4			Sox2			Klf4			c-Myc			Nanog			Lin28		
						
Variables	High n=19	Low n=38	P-value	High n=13	Lo n=44	P-value	High n=7	Low n=50	P-value	High n=21	Low n=36	P-value	High n=13	Low n=44	P-value	High n=28	Low n=29	P-value
Gender																		
Male	16	32	1.0	9	39	0.09	4	44	0.04	19	29	0.32	10	38	0.41	21	27	0.06
Female	3	6		4	5		3	6		2	7		3	6		7	2	
Age (years)																		
≤50	8	23	0.19	3	28	0.01	3	28	0.51	13	18	0.38	5	26	0.19	12	19	0.09
>50	11	15		10	16		4	22		8	18		8	18		16	10	
HBsAg																		
Positive	1	2	1.0	1	2	0.66	0	3	0.51	0	3	0.17	0	3	0.33	2	1	0.53
Negative	18	36		12	42		7	47		21	33		13	41		26	28	
Liver cirrhosis																		
Presence	2	4	1.0	3	3	0.09	2	4	0.10	1	5	0.28	1	5	0.71	3	3	0.96
Absence	17	34		10	41		5	46		20	31		12	39		25	26	
AFP (*μ*g/l)																		
Positive >20	6	12	1.0	2	16	0.15	1	17	0.29	7	11	0.83	4	14	0.94	9	9	0.93
Negative <20	13	26		11	28		6	33		14	25		9	30		20	19	
Tumor number																		
Single	12	30	0.20	8	34	0.26	5	37	0.89	14	28	0.36	10	32	0.76	21	21	0.83
Multiple	7	8		5	10		2	13		7	8		3	12		7	8	
Tumor size^a^ (cm)																		
<5	10	18	0.71	4	24	0.02	4	24	0.65	11	17	0.71	7	21	0.70	10	15	0.03
>5	9	20		9	20		3	26		10	19		6	23		18	11	
Vascular invasion																		
Yes	6	10	0.17	5	11	0.34	4	12	0.02	10	6	0.01	6	10	0.10	7	9	0.60
No	13	28		8	33		3	38		11	30		7	34		21	20	
Tumor encapsulation																		
Complete	11	16	0.26	5	22	0.34	5	22	0.17	13	14	0.09	9	18	0.07	13	14	0.90
Incomplete	8	22		8	22		2	28		8	22		4	26		15	15	
Differentiation																		
I–II	14	35	0.12	11	38	0.87	5	44	0.03	18	31	0.97	10	39	0.26	24	25	0.96
III–IV	5	3		2	6		2	6		3	5		3	5		4	4	
TNM stage																		
I	8	16	0.85	4	20	0.62	3	21	0.49	9	15	0.21	5	19	0.56	10	14	0.341
II	9	16		7	18		4	21		7	18		5	20		15	10	
III	2	6		2	6		0	8		5	3		3	5		3	5	

Data are given as the median.

a Diameter of multiple tumors was calculated as the sum of the size of every single tumor. HBsAg, hepatitis B surface antigen; AFP, α-fetoprotein.
